# Mammary Gland Specific Knockdown of the Physiological Surge in Cx26 during Lactation Retains Normal Mammary Gland Development and Function

**DOI:** 10.1371/journal.pone.0101546

**Published:** 2014-07-02

**Authors:** Michael K. G. Stewart, Isabelle Plante, John F. Bechberger, Christian C. Naus, Dale W. Laird

**Affiliations:** 1 Department of Physiology and Pharmacology, University of Western Ontario, London, Ontario, Canada; 2 INRS–Institut Armand-Frappier, Laval, Québec, Canada; 3 Department of Cellular and Physiological Sciences, University of British Columbia, Vancouver, British Columbia, Canada; 4 Department of Anatomy and Cell Biology, University of Western Ontario, London, Ontario, Canada; Emory University School of Medicine, United States of America

## Abstract

Connexin26 (Cx26) is the major Cx protein expressed in the human mammary gland and is up-regulated during pregnancy while remaining elevated throughout lactation. It is currently unknown if patients with loss-of-function Cx26 mutations that result in hearing loss and skin diseases have a greater susceptibility to impaired breast development. To investigate if Cx26 plays a critical role in mammary gland development and differentiation, a novel Cx26 conditional knockout mouse model was generated by crossing Cx26^fl/fl^ mice with mice expressing Cre under the β-Lactoglobulin promoter. Conditional knockdown of Cx26 from the mammary gland resulted in a dramatic reduction in detectable gap junction plaques confirmed by a significant ∼65-70% reduction in Cx26 mRNA and protein throughout parturition and lactation. Interestingly, this reduction was accompanied by a decrease in mammary gland Cx30 gap junction plaques at parturition, while no change was observed for Cx32 or Cx43. Whole mount, histological and immunofluorescent assessment of breast tissue revealed comparatively normal lobuloalveolar development following pregnancy in the conditionally knockdown mice compared to control mice. In addition, glands from genetically-modified mice were capable of producing milk proteins that were evident in the lumen of alveoli and ducts at similar levels as controls, suggesting normal gland function. Together, our results suggest that low levels of Cx26 expression throughout pregnancy and lactation, and not the physiological surge in Cx26, is sufficient for normal gland development and function.

## Introduction

Breast morphogenesis is unique to other organ systems in that the majority of its development occurs after birth. The progression towards a terminally differentiated gland requires the onset of pregnancy in which alterations in hormones and cell-cell signaling result in the growth and increased branching of ducts, along with the differentiation of alveolar cells to form the mature milk-secretory organ [1]. Weaning leads to the regression and remodeling of the breast to a virgin gland state in a process known as involution. The mammary gland of mice is comparable to that of humans and has frequently been used as a model to study mammary gland development [2]. Although much is known about the hormonal control of mammary gland throughout development, less is known about local communication such as the gap junction proteins connexins (Cxs) [3], [4]. Importantly, gap junctions have been shown to play a role in coordinating cellular tissue responses downstream of hormonal/paracrine stimuli suggesting that dysregulation of Cxs may alter hormonally controlled organ development and function [5].

Six connexin subunits oligomerize to form a hemichannel (or connexon) capable of permitting the exchange of small molecules between the intracellular and extracellular environment. More commonly, connexons from one cell dock with connexons from an adjacent cell to allow for direct gap junctional intercellular communication (GJIC) [6]. Two connexins, Cx26 and Cx43, were shown to be expressed within the breast of humans in which Cx26 is the only connexin localized to the luminal epithelial cells [7]. In mice, Cx26 is expressed at low levels at all stages of mammary gland development and is dramatically upregulated during pregnancy to become the most predominantly expressed connexin within the mammary gland [3], [8]. As such, Cx26 has previously been suggested to play a role in coordinating gland development prior to secretory activation as well as having a possible role in maintaining normal tissue homeostasis and differentiation of the non-pregnant mammary gland [3]. In addition, luminal cells in the mouse mammary gland also express Cx32 and Cx30, which are able to form heteromeric/heterotypic channels with Cx26 during pregnancy and lactation. Heteromeric/heterotypic channels alter the properties and permeabilities of luminal gap junction channels, although the transjunctional molecular exchange through these junctional networks remains unclear [9], [10].

Homozygous knockout mice have been unsuccessful in determining the role of Cx26 in the mammary gland. Cx26^−/−^ mice die *in utero* as a result of placental defects rendering examination of the mammary gland impossible [11]. Similarly, the generation of Cx26^KI32^ mice die embryonically as a result of severe lymphedemas [12]. To overcome this, two conditionally ablated mice using the Cre-*loxP* system were developed to assess the role of Cx26 in the mammary gland [13]. Cx26^fl/fl^ mice crossed with mice expressing the Cre transgene under a whey acidic protein (WAP) promoter (referred to as Cx26^fl/fl^;WC) or the mouse mammary gland tumour virus (MMTV) promoter (referred to as Cx26^fl/fl^;MC) were generated. The MMTV promoter expresses Cre recombinase in the mammary gland prior to birth while the WAP promoter initiates the expression of Cre in the later stages of pregnancy [14]. Interestingly, Cx26^fl/fl^;MC mice showed reduced lobuloalveolar development compared to controls due to an increase in apoptosis while no change was observed in Cx26^fl/fl^;WC mice. As a result, pups from Cx26^fl/fl^; MC dams were more likely to die before being weaned, likely from starvation [13]. It was suggested that Cx26 during early pregnancy plays a critical role in epithelial cell survival and that loss of Cx26 may result in abnormal lactation [13].

However, two discrepancies exist that highlight the need for further studies to evaluate the function of Cx26 in mammary gland development and function. First, Yuan et al. (2011) recently found that control MMTV-Cre mice developed by the Hennighausen laboratory, as the founding MMTV-Cre line A mice had impaired mammary gland developmental defects, cautioning the use of this Cre mouse line in mammary gland development studies [15]. It is unknown which MMTV-Cre mouse line was used by Bry et al (2004) and whether such a concern existed in their study [13]. However pregnant MMTV-Cre line A mice were found to develop with reduced lobuloalveolar formation at the onset of lactation as a result of increased apoptosis, while virgin glands developed normally, results consistent with the findings in the Cx26^fl/fl^;MC mouse line [13], [15]. As a result, additional studies are needed to further evaluate the role of Cx26 in mammary gland development and function. Secondly, a human population of patients exist with systemic dysregulated Cx26 expression and loss of Cx26 channel function that have never been reported to have altered breast function. Over 90 mutations in the *GJB2* gene that encodes Cx26 give rise to both syndromic and non-syndromic deafness that accounts for approximately half of congenital cases of hearing impairments [16]. Mutations in *GJB2* may result in both autosomal dominant and recessive loss of function mutations, of which the most frequent recessive mutations may lead to considerable, if not total ablation of Cx26 channel function [17]. Interestingly, despite an incidence rate of this mutation similar to that of cystic fibrosis, there are no reports of breast feeding problems in the deaf community suggesting that Cx26 may not be essential for normal gland function [16], [18].

Thus, in order to further evaluate the role of Cx26 in the mammary gland, we developed a novel Cx26 conditional knockdown mouse model in which β-lactoglobulin (BLG)-Cre mice were crossed with Cx26^fl/fl^ mice. The resulting mice were found to have greatly reduced Cx26 expression in the mammary gland yet retained normal gland development, differentiation and function. These findings were even more remarkable given that cross-talk mechanisms cause a co-regulated reduction in Cx30. Taken together, these findings strongly suggest that mammary gland function can proceed normally in the absence of the physiological surge in Cx26 that occurs during pregnancy and in the presence of a substantial loss of gap junctional exchange of signalling molecules.

## Methods

### Animals

BLG-Cre mice [19] were crossed with Cx26^fl/fl^ mice [20] to produce BLG-Cre;Cx26^fl/fl^ mice (Cx26 knockdown mice). All mice were genotyped for the expression of the Cre transgene (data not shown). In addition, BLG-Cre mice were crossed with WT mice showing no evidence of a lactation defect (data not shown). Four virgin, pregnant d9.5 and pregnant d12.5, as well as eight Cx26 knockdown mice at parturition, d4 of lactation and d2 of involution, along with Cx26^fl/fl^ control mice at identical timepoints, were sacrificed using CO_2_ and O_2_. Thoracic mammary glands were removed and either stored at −80°C (right side) or cryo-embedded (left sided glands), while inguinal mammary glands were either fixed in 10% neutral buffered formalin and then embedded in paraffin (right sided glands) or processed for whole mounting (left sided glands). Mice were arbitrarily numbered to allow experiments to be performed by a process that was blinded to the investigator. The protocol was approved by the Committee on the Ethics of Animal Experiments of the University of Western Ontario (206-101-10) and the University of British Columbia (A11-0170) and the Canadian Council of Animal Care.

### Real-Time PCR

Total mRNA was isolated from the tissues using TRIzol (Invitrogen, Burlington, ON) and purified using RNeasy mini-kit (Quiagen, Mississauga, ON) following the manufacturers' instructions. cDNA were generated from isolated mRNA using RevertAid First Strand cDNA Synthesis Kits (Fermentas, Burlington, ON) and then subjected to specific amplification using SsoFast EvaGreen Supermix (Bio-rad, Mississauga, ON) using Cx26 (5′ TCCGCATCATGATCCTCGTG 3′; 5′ CCCAGAGCCGGATGTGA 3′), Cx30 (5′ GCCGAGTTGTGTTACCTG 3′;5′ GCATTCTGGCCACTATCTGA 3′), Cx32 (5′ CTTGCTCAGTGGCGTGAATC 3′; 5′ CGGCTGGAGGGTGTTACAG 3′), Cx43 (5′TATGACAAGTCCTTCCCCAT 3′; 5′ TGATTTCAATCTGCTTCAGG 3′) and B2M (5′ CCCACTGAGACTGATACATACGC 3′; 5′ GGTTCAAATGAATCTTCAGAGCAT 3′) primers, with a TM at 55°C (N = 4).

### Western blot analysis

Mammary gland tissues were homogenized in lysis buffer and subjected to Western blot analysis, as described previously [21], [22]. Membranes were immunoblotted with the following primary antibodies: mouse anti-Cx26 (C14523, Lifespan, Seattle, WA, 1 µg/ml), rabbit anit-Cx30 (71-2200, Invitrogen, Burlington, ON, 0.25 µg/ml), rabbit-anti-Cx32 (C3470, Sigma-Aldrich, Oakville, ON, 0.1 µg/ml), rabbit anti-Cx43 (C6219, Sigma-Aldrich, Oakville, ON, 0.1 µg/ml), goat anti-WAP and goat anti-β-casein (sc-14832, sc-17971, Santa Cruz Biotechnology, Dallas, TX, 0.2 µg/ml), mouse anti-glyceraldehyde-3-phosphate dehydrogenase (GAPDH) (MAB374, Millipore, Billerica, MA, 2 µg/ml) and rabbit anti-GAPDH (14C10, Cell Signaling, Danvers, MA, 1∶1000). Bound primary antibody was detected using the following fluorescence-conjugated secondary antibodies; Alexa 680-conjugated goat anti-rabbit, Alexa 800-conjugated goat anti-mouse and Alexa 680-conjugated donkey anti-goat (Molecular Probes, Eugene, OR, 0.2 µg/ml) followed by visualization and quantification using the Odyssey Infrared Imaging System (Li-Cor Biosciences, Lincoln, NE). Protein levels were determined by normalization to the Western blot band intensities of GAPDH (N≥6).

### Immunofluorescence

Paraffin-embedded sections (6 µm) were deparaffinized in xylene and rehydrated in descending concentrations of ethanol baths, microwaved for 5 min (80%) in antigen-retrieval solution (1∶50, Vector Labs) and put into 0.01 M Tris/1 mM EDTA buffer (pH 9.0) at 90–95°C for 30 min, as previously described [22]. Cryo-sections (7 µm) were fixed with 10% neutral buffered formalin. Paraffin and/or cryosections were blocked with 0.1% Triton X-100 and 3% BSA in PBS for 60 minutes at room temperature. Sections were then incubated with the following primary antibodies: rabbit anti-Cx26 (51-2800, Invitrogen, Burlington, ON, 2.5 µg/ml), rabbit anti-Cx30 (71-2200, Invitrogen, Burlington, ON, 2.5 µg/ml), rabbit-anti-Cx32 (C3470, Sigma-Aldrich, Oakville, ON, 1.0 µg/ml), rabbit anti-Cx43 (C6219, Sigma-Aldrich, Oakville, ON, 1.0 µg/ml), mouse pan-Cytokeratin (ab7753, Abcam, Cambridge, MA, 4 µg/ml), mouse anti-Proliferating Cell Nuclear Antigen (PCNA) (M-0879, Dako, Burlington, ON, 0.5 µg/ml), goat anti-WAP (sc-14832, Santa Cruz Biotechnology, Dallas, TX, 2 µg/ml) and Wheat Germ Agglutinin-633 (WGA) conjugate (W-21404, Invitrogen, Burlington, ON, 1∶400). Primary antibody was visualized by incubating sections with secondary antibodies: Alexa480-conjugated goat anti-rabbit, Alexa480-conjugated goat anti-mouse, Alexa555-conjugated anti-mouse secondary antibody, (Invitrogen, Burlington, ON, 0.5 µg/ml) or anti-goat Texas Red (Jackson ImmunoResearch Laboratories, Baltimore, PA, 1∶100). Hoechst stain was used to visualize nuclei prior to mounting. Immunolabeled sections were imaged (5–10 images per sample) using a Leica DM IRE2 inverted epifluorescence microscope equipped with Openlab 5.5.3/Velocity 6.3.0 imaging software or a Zeiss LSM 510 inverted confocal microscope (N≥6). For cytokeratin area quantification, green only and blue only fluorescent images at 20X magnification were converted to binary using ImageJ and the pixel area was measured. Graphs represent the mean ratio of green fluorescent area (cytokeratin) to blue fluorescent area (nuclei). For PCNA quantification, the number of PCNA positive cells per 20X field was quantified. In addition, the blue only fluorescent images at 20X magnification were converted to binary using ImageJ and the pixel area was measured. Graphs represent the mean ratio of the number of PCNA positive cells to blue fluorescent pixel area (nuclei).

### Whole mounting

As previously described [23], the left inguinal mammary glands were excised, spread on glass slides and fixed in Carnoy's fixative (100% EtOH, chloroform, glacial acetic acid; 6∶3∶1) for 4 h at room temperature. Mammary glands were washed in 70% EtOH for 15 min, gradually rehydrated in water, and stained in carmine alum (2% carmine and 5% aluminum potassium sulfate in water) overnight at room temperature. Tissues were then gradually dehydrated through serial ethanol baths and cleared in xylene overnight. Mammary glands were kept in methyl salicylate until images were captured with a numeric camera (Cybershot, Sony) and a SteREOLumar V12 microscope (Zeiss) (N≥4).

### Histology

Paraffin-embedded mammary gland sections (6 µm) were deparaffinized in xylene for 10 min, rehydrated in descending grades of ethanol baths, and stained with 1% Harris's haematoxylin and 1% eosin. Sections were dehydrated in ascending grades of ethanol and xylene baths and mounted with Cytoseal (Richard-Allan Scientific). Qualitative histological analysis was performed by imaging several arbitrary areas per 20× field of view using a Leica DM IRE2 inverted epifluorescence microscope equipped with a ProgRes C5 camera (Jenoptik) and ProgRes Mac CapturePro 2.7.6 imaging software (N = 8).

### Evaluation of apoptosis

Apoptotic cells were stained using a commercial kit for TUNEL assay (Apoptag, Chemicon International, Temecula, CA) following the manufacturer's instructions. Slides were treated with 0.5% TritonX-100 in PBS. 5–10 random fields per mouse were evaluated in which the ratio of the number of apoptotic cells to the pixel area of nuclei were quantified using ImageJ software (National Institutes of Health, Bethesda, MD) for each 20X field (N = 5).

### Statistical analysis

All statistical analyses were performed using GraphPad Prism version 4.03 for Windows. A Student's unpaired t-test or a one-way ANOVA test was used in which a p<0.05 was considered significant. Values are presented as means ±S.E.M.

## Results

### Conditionally ablated Cx26 in the mammary gland

**Figure 1:**
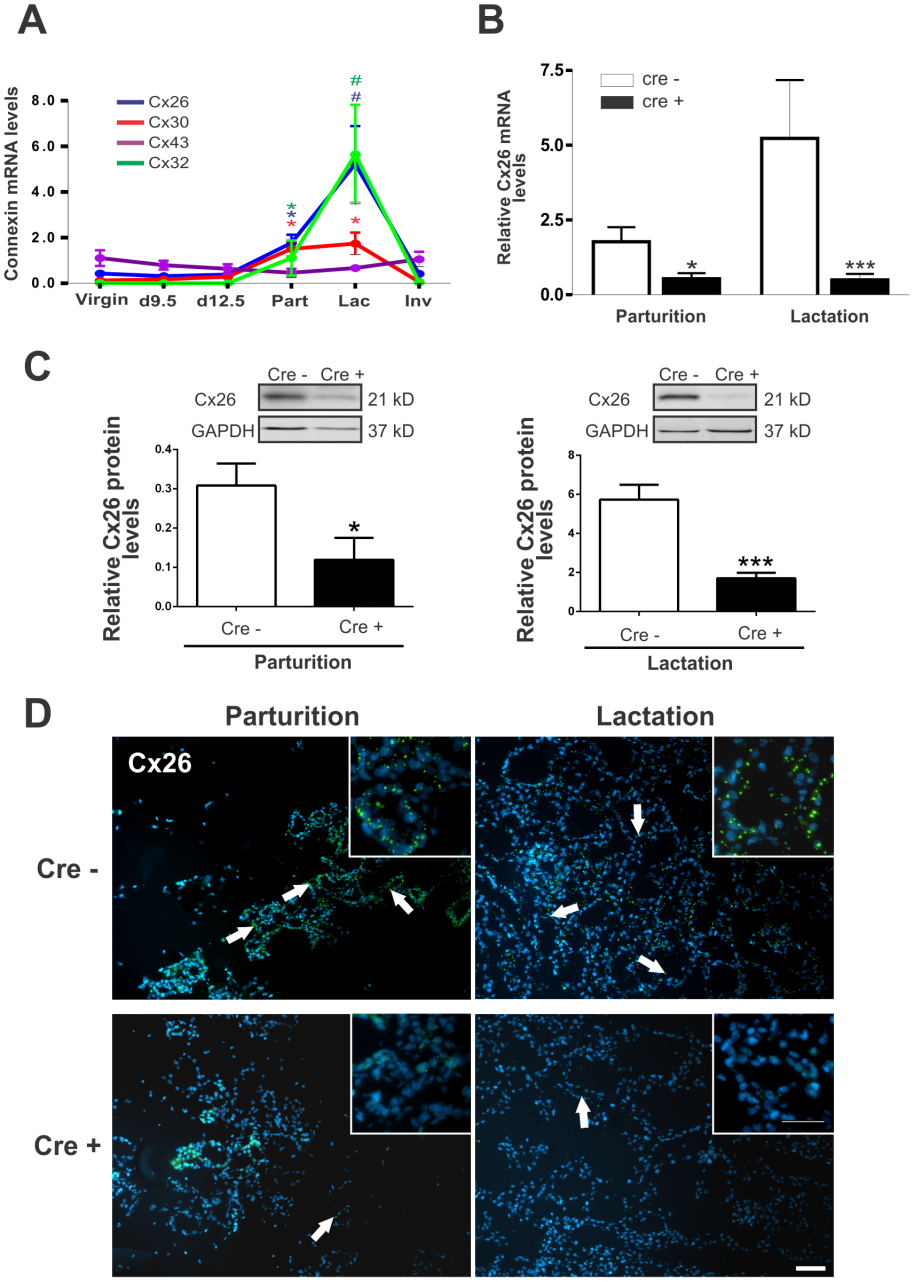
BLG-Cre; Cx26^fl/fl^ mice exhibit a dramatic reduction in Cx26. (A) Real-time PCR analysis of wild-type mice revealed that Cx26, Cx32 and Cx30 are upregulated at parturition and lactation. (B, C) Real-time PCR and Western blot analysis of mammary glands from control (open columns) and Cre-treated (solid columns) mice revealed a dramatic reduction in Cx26 mRNA and protein levels at partition and lactation. *p<0.05, ***p<0.001. Values are mean levels ±SEM. N≥4. (D) Immunofluorescence for Cx26 revealed a decrease in Cx26 gap junctions (arrows) at parturition and lactation in Cre-treated mice compared to control mice. Hoechst staining denotes the nuclei. N = 6. Scale Bar  = 50 µm.

In the mammary gland, Cx26, as well as other luminal Cx30 and Cx32, expression is dramatically upregulated at parturition and remains elevated throughout lactation (Figure 1A, [10]). To assess the efficiency of the Cx26 knockdown we performed real-time PCR analysis of Cx26 which revealed a significant decrease, but not complete ablation, of Cx26 mRNA at parturition and lactation (Figure 1B). Consistent with these results, Western blot and immunofluorescent analysis revealed a significant ∼65–70% decrease in Cx26 protein levels (Figure 1C) that correlated with a decrease in Cx26 gap junction plaques within the mammary epithelium of Cx26 knockdown mice at parturition and lactation (Figure 1D). As expected, since BLG promoter is not activated before mid-pregnancy (day 10 of pregnancy), no differences were observed in other stages (Figure S1). Together, residual Cx26 expression suggests that Cx26 knockdown mice maintain low basal expression of Cx26 throughout the pregnancy and lactation phases of mammary gland development while the typical physiological surge in Cx26 was eliminated.

**Figure 2:**
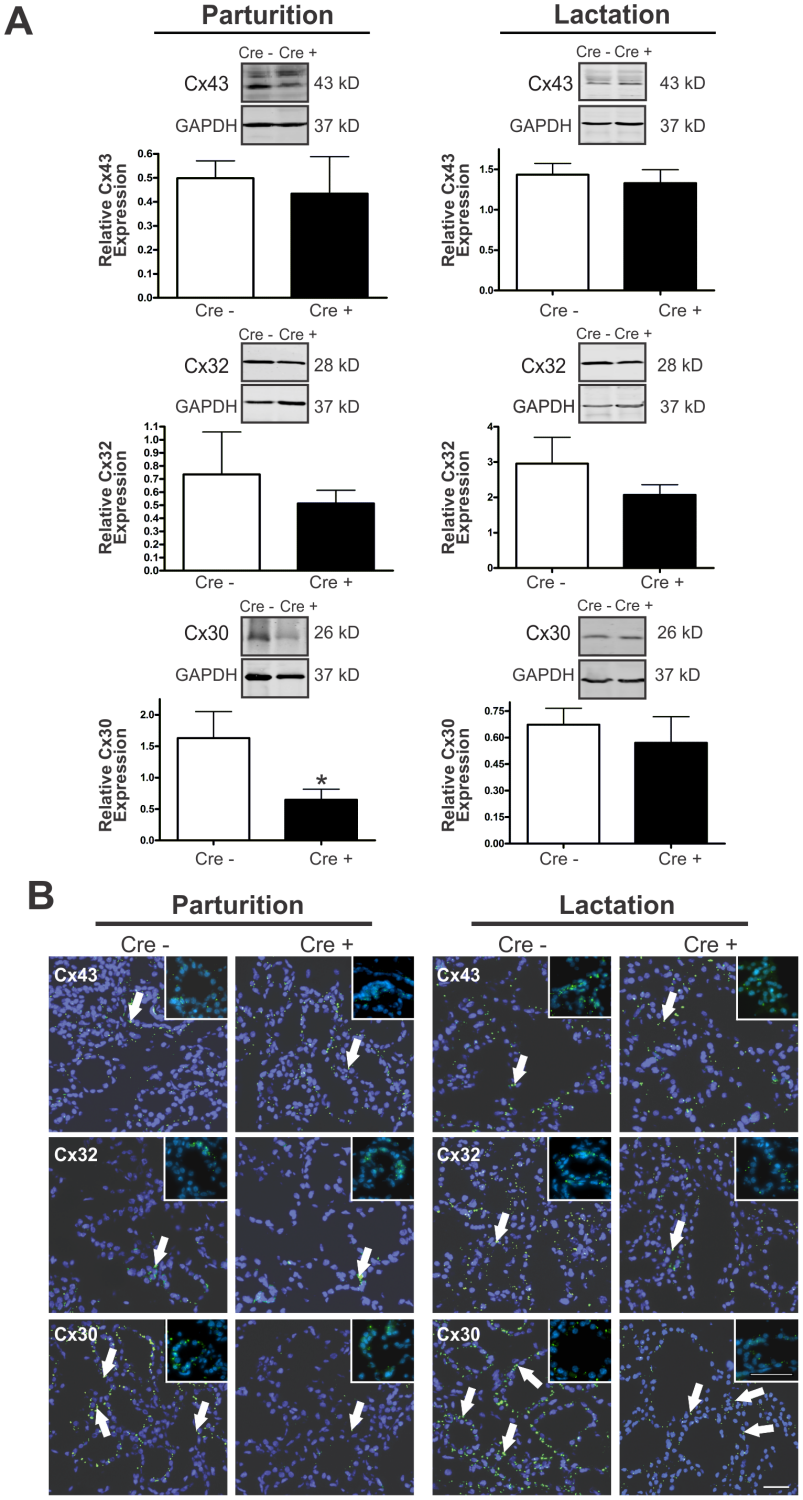
Lactating mammary glands of Cx26 knockdown mice exhibited a cross-talk reduction in Cx30 at parturition. (A) Western blot and (B) immunofluorescent analysis of mammary glands from control and Cre-treated mice at parturition revealed a significant decrease in Cx30 expression and a reduction in Cx30 gap junction plaques, while no change was observed in either Cx43 or Cx32. Arrows denote connexin plaques. *p<0.05. Values are mean levels ±SEM. N≥6. Hoechst staining denotes the nuclei. Scale Bars = 50 µm.

To assess the status of other connexins co-expressed within the mammary gland in response to a reduction in Cx26, Western blot and immunofluorescent analysis of Cx43, Cx32 and Cx30 were evaluated at parturition and lactation. Protein expression and localization of Cx43 and Cx32 were similar in the mammary glands of lactating Cx26 knockdown mice compared to control mice (Figure 2A, 2B). Interestingly, our assays revealed a significant decrease in Cx30 expression to about ∼40% of control and a significant reduction in Cx30 gap junction plaques in lactating Cx26 knockdown mice compared to control (Figure 2A, 2B, S2A). Qualitative immunofluorescent analysis of Cx30 gap junctions at lactation showed that Cx30 gap junctions appeared dramatically smaller in Cx26 knockdown mice compared to control mice despite a similar number of overall Cx30 gap junction plaques (Figure 2B, S2B). Therefore, the Cx26 knockdown mice lacked the physiological surge in both Cx26 and Cx30 that are typically observed in the mammary gland during pregnancy.

### Cx26 knockdown mice retain normal mammary gland development throughout pregnancy

**Figure 3:**
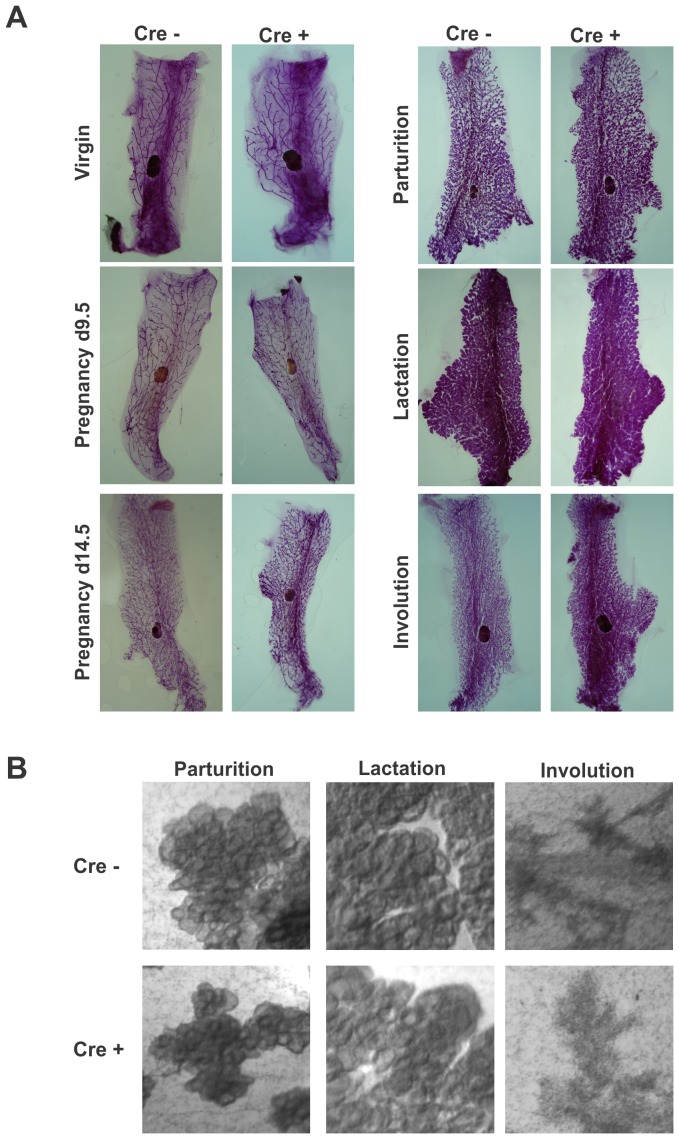
Mammary glands from Cx26 knockdown mice at all stages of gland development have similar gross gland architecture. (A) Whole mount analysis of developing and differentiating mammary glands from control and Cx26 knockdown mice revealed similar ductal and alveolar structure as assessed with carmine alum staining. (B) High (25X) magnification of whole mounts revealed developed alveoli in lactating Cx26 knockdown mice and alveolar turnover in involuting mice similar to controls. N≥4.

To assess whether a reduction in Cx26 gap junction plaques affects the development of the mammary gland during the pregnancy cycle, whole mount analysis was assessed in the mammary glands of virgin, pregnancy day 9.5, pregnancy day 12.5, at parturition, lactating and involuting mice. Adult virgin mammary glands of genetically-modified mice contained an expansive epithelial ductal network embedded within a developed mammary fat pad similar to control mice (Figure 3A). Following the onset of pregnancy, whole mount assessment revealed increased ductal branching and lobuloalveolar structures in the glands of Cx26 knockdown mice similar to control mice suggesting normal timing of alveolar development. In accordance, mammary glands of the genetically-modified mice at all stages of development following pregnancy were similar to Cx26^fl/fl^ control mice with developed alveoli that fully encompassed the mammary gland by early lactation and comparable alveolar turnover during involution (Figure 3A, 3B).

**Figure 4:**
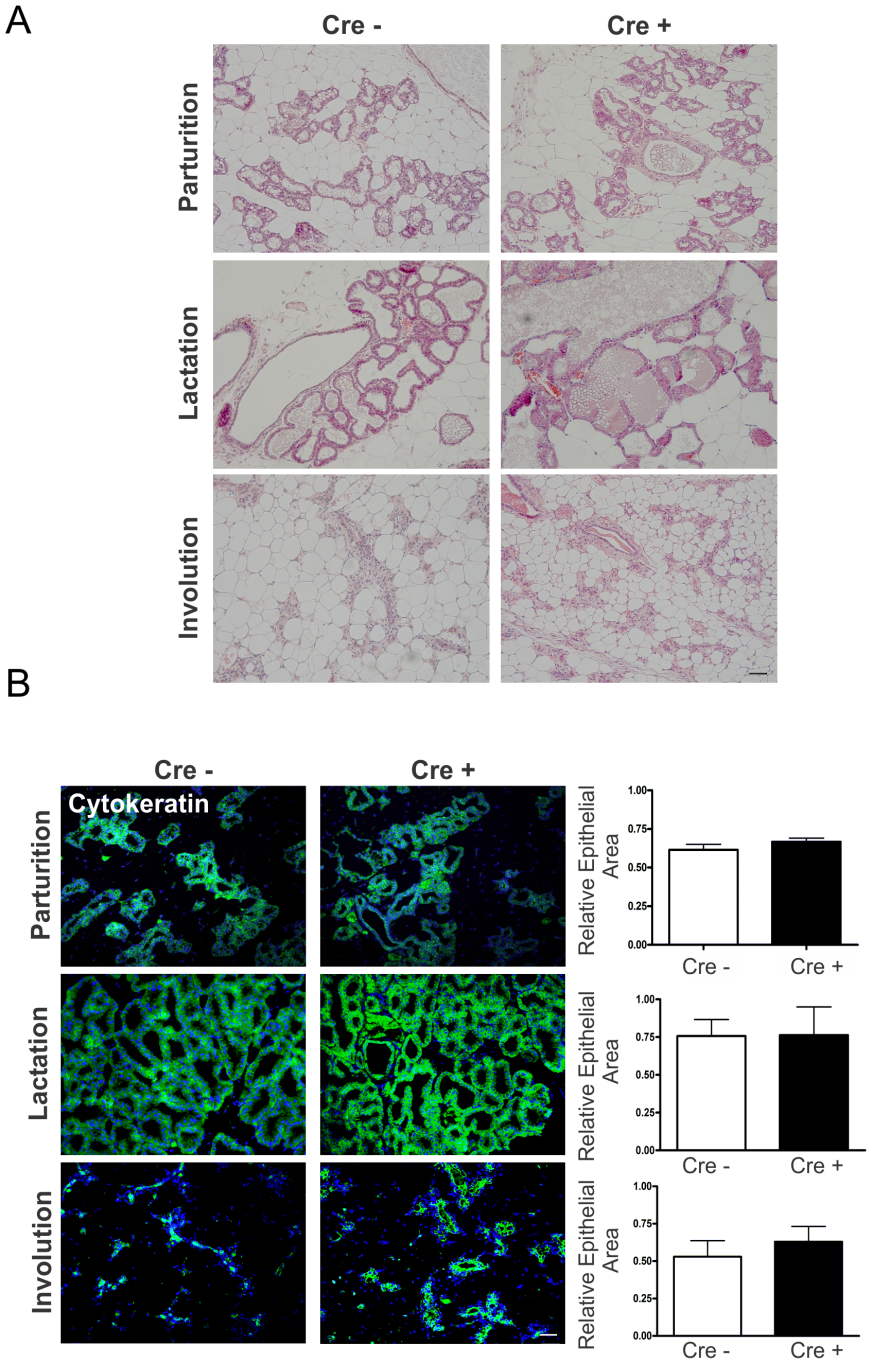
Mammary glands of Cx26 knockdown mice have similar epithelial development. (A) Haematoxylin and eosin staining of lactating and involuting mammary glands revealed similar histology between genetically-modified and control mice. Scale bars = 100 µm. (B) Labeling for the epithelial marker, cytokeratin, using an anti-pan-cytokeratin antibody revealed similar levels of labeling in control and genetically-modified mice. Values represent the mean positive-staining pixel area (green) relative to the pixel area of the nuclei (blue) per 0.18 mm^2^±S.E.M. N = 8. Hoechst staining denotes the nuclei. Scale Bars = 50 µm.

Since whole mount analysis can conceal alveoli changes due to the density of alveoli, histological analysis of H&E stained sections were performed on control and genetically-modified mice. These studies revealed no qualitative differences in the density of alveoli and ducts throughout the mammary gland regardless of the stage of development after pregnancy (Figure 4A). In addition, to quantitatively evaluate whether conditionally reduced Cx26 led to defects in alveologenesis, mammary gland sections at parturition, lactation and involution were immunolabelled with the epithelial marker pan-cytokeratin. Ten arbitrary images were taken of each slide and the area of cytokeratin over the area of the nuclei was quantified. Quantification revealed no significant difference in cytokeratin labelling between Cx26 knockdown mice and Cx26^fl/fl^ control mice at parturition, lactation and involution suggesting normal epithelial turnover throughout lactation and involution (Figure 4B).

**Figure 5:**
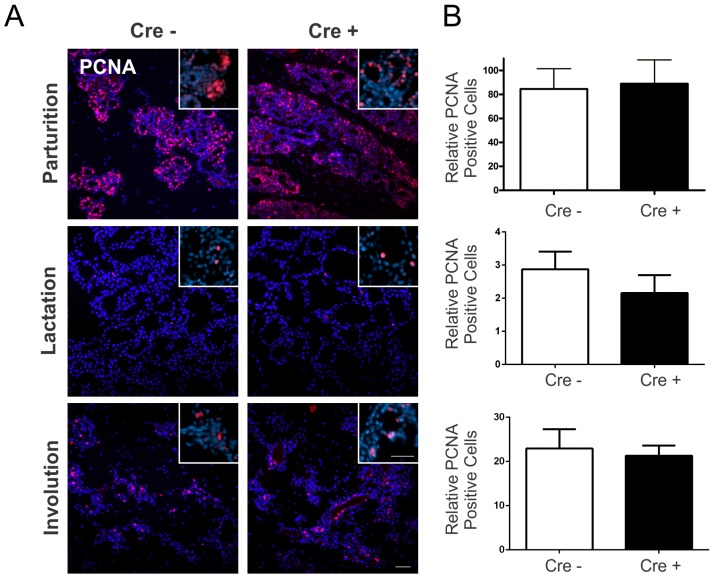
The mammary gland of Cx26 knockdown mice has unaltered cell proliferation at parturition, lactation and involution. (A) Assessment of PCNA labeling (red) revealed no change between Cre-treated and control mice. (B) Values represent the mean number of positive cells (inserts, red) relative to the pixel area of the nuclei (blue), divided by a factor of 1×10^−9^, per 0.18 mm^2^±S.E.M. N = 8. Hoechst staining denotes the nuclei. Scale bars = 50 µm.

Previously, Lee et al. (1992) demonstrated that Cx26 mRNA was upregulated in the late G1 and early S phase of normal mammary epithelial cells suggesting that Cx26 may have a role in luminal cell proliferation [24]. To assess whether Cx26 regulates proliferation through gland development, Cx26 knockdown mice at parturition, lactation and involution were immunolabelled with the proliferation marker PCNA and compared to Cx26^fl/fl^ mice. Quantification revealed no significant difference in the number of PCNA positive cells at all-time points in Cx26 knockdown mice compared to controls suggesting that the physiological surge in Cx26 is not critical in regulating proliferative mechanisms of gland development during pregnancy (Figure 5).

**Figure 6:**
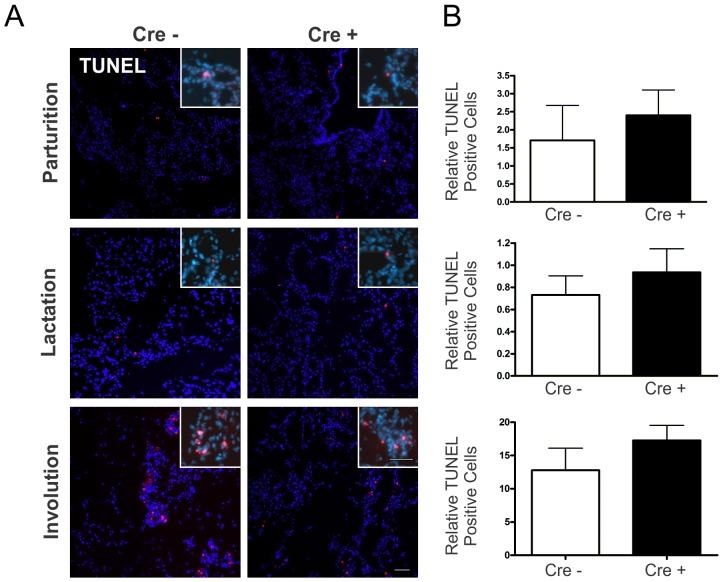
Programmed cell death is unaltered in lactating and involuting mammary glands form Cx26 knockdown mice. (A) Evaluation of TUNEL positive (red) cells revealed no difference between genetically-modified mice and control. (B) Values are mean number of positive-cells (inserts) relative to the pixel area of the nuclei, divided by a factor of 1×10^−9^, per 0.18 mm^2^±S.E.M. N = 5. Hoechst staining denotes the nuclei. Scale bars = 50 µm.

As Cx26 has previously been implicated in regulating epithelial cell survival during the early phase of pregnancy, mammary glands of Cx26 knockdown mice were assessed for changes in apoptosis using a TUNEL assay [13]. Quantification of TUNEL positive cells showed relatively low numbers of apoptotic cells at parturition and lactation and increased apoptosis during involution in Cx26 knockdown mice similar to Cx26^fl/fl^ mice (Figure 6). These results suggest that conditional knockdown of Cx26 during early pregnancy does not alter epithelial cell survival. Collectively, our results suggest that basal Cx26 expression is sufficient to retain normal mammary gland development.

### Cx26 knockdown mice have normal mammary gland lactation

**Figure 7:**
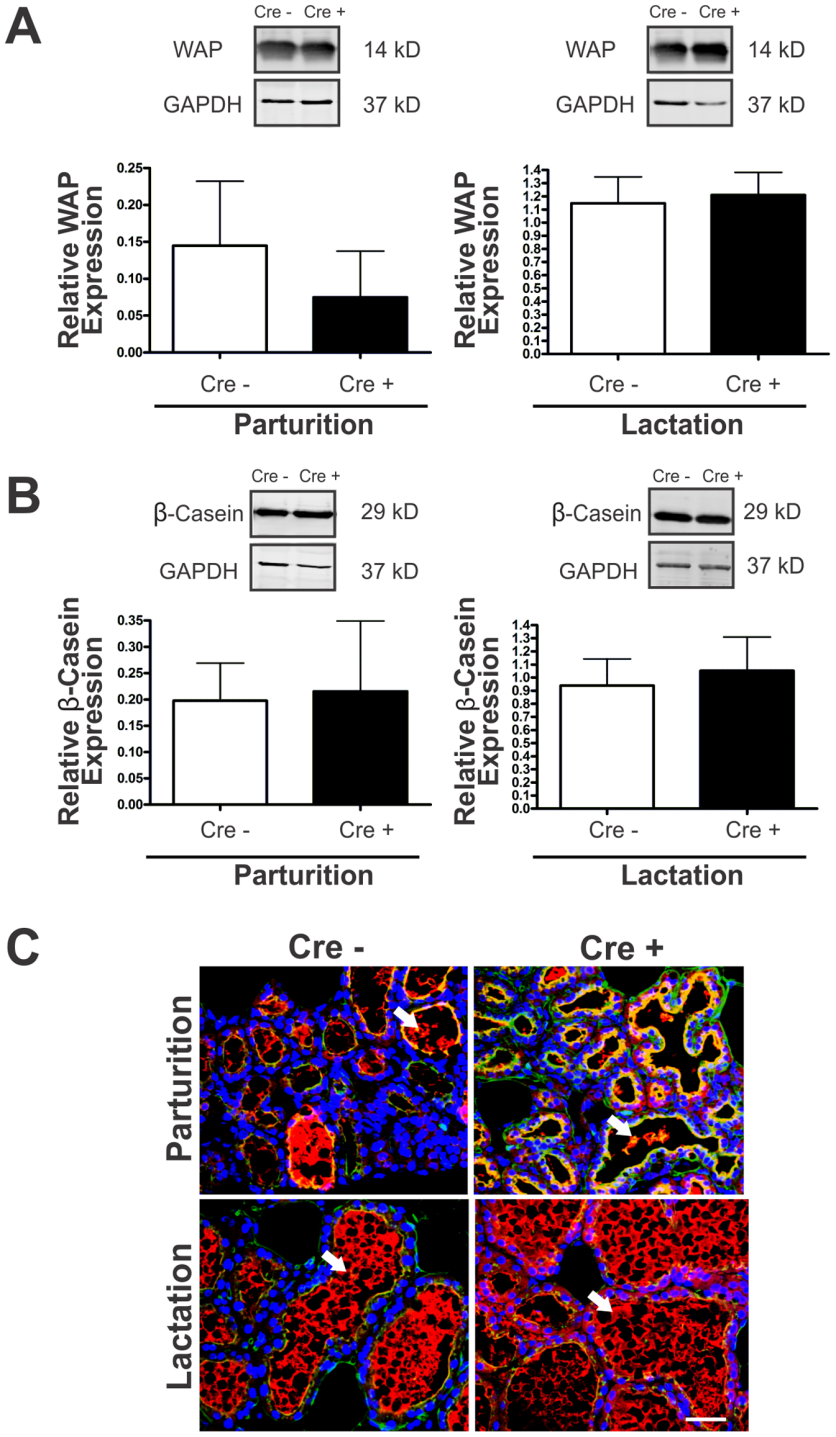
Similar to control mice, lactating Cx26 knockdown mice can produce and secrete milk. (A, B) Western blot analysis revealed similar WAP and β-casein expression levels at parturition and lactation of control and genetically-modified mice. Values are mean levels ±SEM. N = 8. (C) Immunofluorescent analysis revealed secreted WAP (red, arrows) within the lumen of ducts and alveoli outlined with wheat germ agglutinin (green). N = 4. Hoechst staining denotes the nuclei. Scale bars = 50 µm.

To determine the functional state of the gland, Western blot analysis of 2 common milk proteins, WAP and β-casein, were performed. Our data revealed similar expression in lactating Cx26 knockdown and control mice (Figure 7A, 7B). As the Western blot analysis is a measure of both secreted and non-secreted milk proteins, immunofluorescence of WAP within the ducts (apical epithelium marked with WGA) was used as a measure of milk secretion. Evidence of secreted milk within the alveoli of Cx26 knockdown and control mice was observed (Figure 7C). In addition, postnatal day 18 pup weights from both Cx26 knockdown and WT dams did not show any significant differences as well as similar litter sizes (data not shown) suggesting that pups survive to weaning age and do not die of starvation. During subsequent 2^nd^ and 3^rd^ pregnancies, no differences were seen in the litter weight sizes or health of the pups. Thus, a reduction in Cx26 did not affect later pregnancies as well. These findings are consistent with the whole mounting, histological and immunolabeling data further supporting the premise that the absence of the physiological surge in Cx26 during pregnancy does not result in an overt developmental or functional defect in the mammary gland in Cx26 knockdown mice.

## Discussion

In the present study we use a novel, conditional Cx26 knockdown mouse model to evaluate the biological role of the Cx26 surge seen in the mammary gland as mice enter and proceed through pregnancy. In contrast to previous reports, we demonstrated that the absence in the physiological surge in Cx26, resulting in reduced Cx30 upregulation, during pregnancy is not necessary for normal mammary gland development. Furthermore, we showed that low basal levels of Cx26 are sufficient for normal mammary gland function.

### Cx26 knockdown mice as a model to study the role of Cx26 in mammary gland development and function

Conditionally ablated mice have previously been described to study the role of Cx26 in mammary gland development. These mice were engineered to express Cre under the MMTV or WAP promoters that become activated at an early embryonic stage or at the end stages of pregnancy, respectively [13], [14]. However, both of these genetically-modified mice ablate Cx26 from birth or after pregnancy begins but not at the onset of Cx26 upregulation at D8.5 of pregnancy [10]. Thus, in order to assess Cx26's role during pregnancy, we employed a similar Cre-*lox*P targeting strategy using Cre under the BLG promoter as previously described [19]. Cre-mediated recombination following upregulation under the BLG promoter has been reported to become activated at approximately day 10 of pregnancy causing recombination of the floxed allele in 70–80% of the lactating mammary gland [19], [25]. In comparison to the MMTV or WAP promoters, BLG promoter activation is more restricted to the mammary gland with ≤1% recombination reported in other tissues while the MMTV promoter has also been reported in the salivary glands, seminal vesicles and lymphoid cells and the WAP promoter is expressed within the brain [14], [26]. Thus, the BLG promoter driven elimination of Cx26 more precisely mimics the physiological increase in Cx26 [19]. Here we demonstrated that Cx26 knockdown mice had a reduction in the physiological surge in Cx26 during mammary gland development but did not completely ablate Cx26 expression. This reduction in Cx26 was not unlike the Cx26^fl/fl^;MC mice reported by Bry et al (2004) where residual Cx26 may also have be present [13]. Importantly, the expression of Cx26 gap junctions is localized to only select luminal cells in our Cx26 knockdown mice suggesting that the majority of luminal cells may have complete ablation of Cx26.

Downregulation of untargeted connexins in response to specific connexin knockout targeting strategies has been observed in a variety of tissues, including the pancreas and liver of Cx32^−/−^ deficient mice in which Cx26 was also reduced [27], [28]. In our study, we observed a reduction in Cx30 upregulation concordant with a knockout strategy targeting Cx26. Similarly, the conditional ablation of Cx26 in the inner ear of mice expressing Cre under the Sox2 promoter showed a delay in the expression of Cx30 till postnatal d14, unlike in control mice where Cx30 expression was observed as early as postnatal d6 [29]. Our results suggest that cross talk regulation of Cx26 and Cx30 expression is not exclusively restricted to the inner ear. Although not tested, we speculate that the reduction in Cx30 occurs through the regulation of gene transcription similar to that described previously [29], although a post-translational role of Cx26 to stabilize Cx30 containing hemichannels through oligomerization cannot be ruled-out. Alternatively, Cx30 downregulation is also associated with a downregulation in Cx26 as mice generated to express the Cx30^T5M^ mutant have a significant downregulation of Cx26 within the adult cochlea suggesting that Cx26 and Cx30 share reciprocal cross talk mechanisms [30]. Indeed, this regulation between Cx26 and Cx30 was suggested to be mediated through calcium and NF-kB signaling, although it is unknown if a similar mechanism occurs within the mammary gland [31]. Importantly, both calcium and NFkB signaling occur during pregnancy and lactation within the mammary gland [32], [33].

Although the down regulation of Cx26 or Cx32 has previously been reported to reciprocally down-regulate each other [27], this was not observed in the Cx26 knockdown mice used in this study and may reflect the differential timing and possibly distinct mechanisms of Cx26 (upregulated during pregnancy) and Cx32 (upregulated during early lactation) expression within the mammary gland [10]. Similarly, Cx43 expression was not altered in Cx26 knockdown mice compared to control mice which likely reflects their differential localization patterns as Cx26 is expressed mainly in luminal cells while Cx43 is found mainly in the myoepithelium and stromal fibroblasts [3]. Together, Cx26 knockdown mice have acquired a phenotype where Cx26 is reduced in lactating mice with a delay in Cx30 upregulation.

### Mammary gland development is maintained in Cx26 knockdown mice

All exocrine glands have previously been shown to express Cx26 and/or Cx32 gap junctions suggesting that these proteins may be critical in regulating gland development [34]. To date, systemic loss of Cx32 or Cx26 has yet to reveal impaired development of exocrine tissues [35]. However, as in the case of Cx26, this is difficult to assess due to the embryonic lethality of Cx26 mice at embryonic D10.5 [11]. The question then arises as to why Cx26 appeared to play a key role in the exocrine mammary gland lobuloalveolar development which led to impaired lactation in MMTV-Cre mice [13]. Three possibilities exist to help explain this discrepancy. First, it is plausible that because mammary gland development is unique in that most of its development occurs after birth, Cx26 may have a unique role to guide its development [3]. Second, as the use of control MMTV-Cre mice in the Bry et al. study were not reported to assess whether the insertion of MMTV-Cre affected the phenotype of these mice, and given the new finding by Yuan et al (2011) that at least one mouse line of MMTV-Cre mice has mammary gland defects, we cannot rule-out the possibility that conditional targeting strategies using MMTV-Cre mice are less than ideal to examine mammary gland development and function [15]. However, in support of the validity of the findings from MMTV-Cre driven Cx26 knockout mice, these mice showed a clear paucity of alveolar formation by pregnancy d15 compared to control mice while decreased alveoli and ducts were not observed until parturition in MMTV-Cre mice suggesting a similar but distinct phenotype [13], [15]. Finally, it is possible that Cx26 may play an important role in the development of other exocrine glands and that further evaluation of these glands using similar conditional targeting strategies is needed.

Whole mount, histological and immunofluorescent analysis of BLG-Cre driven Cx26 knockdown mice revealed that the physiological surge in Cx26 is not required for mammary gland development or for controlling proliferative or apoptotic mechanisms associated with pregnancy, lactation and the involution cycle of the mammary gland. Our results may suggest one of two possibilities: First, low levels of Cx26 during pregnancy and lactation are sufficient to mediate alveologenesis and gland function. This appears to be the case in terms of Cx43 within the mammary gland, in which the Cx43^I130T/+^ mice that maintained GJIC above a certain threshold was able to retain proper mammary gland development and function not observed in Cx43^G60S/+^ mice with low GJIC [22]. Our study did not evaluate GJIC therefore it is unknown whether a similar observation is observed in the BLG-Cre driven Cx26 knockdown mice. Second, our results may also suggest that Cx26 does not regulate alveologenesis during pregnancy but instead is important earlier in development. As the MMTV promoter is activated embryonically and Cx26 has been found to be expressed at low levels in the virgin mammary gland, it is unknown whether Cx26 plays a role in regulating differentiation of the stem/progenitor cells within the mammary gland prior to pregnancy [14], [21]. Importantly, Cx26 has been reported to be expressed in stem/progenitor cells postnatal hippocampus but it is unknown if Cx26 is expressed in mammary stem/progenitor cells [36]. Regardless of which of these two possibilities is correct, the maintenance of basal levels of Cx26 during pregnancy does not appear to impair lobuloalveolar development of the mammary gland.

### Mammary gland function is maintained in Cx26 knockdown mice

Both Cx26 and Cx32 have been suggested to regulate and fine tune the synthesis and release of factors in exocrine glands, of which Cx32 is thought to play the dominant role [34]. However, Cx32 null mice do not present with a lactation defect suggesting that other connexins such as Cx26 or Cx30 channels, are equally important in regulating mouse mammary gland secretion [3]. Our results suggest that the absence of the physiological surge of Cx26 does not appear to impair the secretory function of the gland in that two of the most common milk proteins had no change in their expression or release into the lumen of acini or ducts. Consistent with this, sufficient milk was produced from lactating dams as no difference in pup death was observed in Cx26 knockdown mice, which may be the result of normal Cx32 expression fulfilling the need for the loss of Cx26 within the mammary gland. Importantly, both Cx32 and Cx26 channels are able to pass similar molecules through GJIC including ATP and IP_3_ [37]. In support of this, Cx32 is upregulated at the onset of lactation and has previously been shown to be insensitive to gating by the osmolyte taurine that is implicated in milk protein synthesis, unlike that of Cx26 homomeric and heteromeric Cx26/32 channels [10]. As a result, loss of the physiological surge in Cx26 does not have an overt impairment on milk production or delivery to pups.

## Conclusions

In humans, Cx26 is the only consistently reported connexin to date to be expressed within the luminal epithelium suggesting that Cx26 is the dominant connexin regulating human mammary gland function [3]. As such, loss of this connexin would presumably result in breast feeding defects as previously reported in MMTV driven Cx26-ablated mice. Interestingly, a population of patients exist with mutations in the *GJB2* gene that encodes Cx26 representing a large population base with systemic and impaired Cx26 channels that result in deafness and skin diseases [17]. Although no study to date has specifically evaluated a relationship between loss of function mutations in *GJB2* patients and breast feeding, it is interesting that in the face of such a high prevalence of people with these mutations resulting in deafness, there are no reports of lactation defects within the deaf community [18]. Two possibilities arise to explain this discrepancy. First, that breast feeding defects occur within hearing impaired mothers expressing Cx26 mutants but are not reported. Second, Cx26 is not a critical regulator of epithelial cell survival and that maintenance of low levels of Cx26 is sufficient for breast feeding. Our mouse model supports the latter and suggests that mothers with *GJB2* mutations that maintain Cx26 levels above 30% will not develop breast feeding defects.

In summary, our novel mouse model suggests that in the absence of the physiological surge in Cx26 mammary gland development and function are retained within the mammary gland of mice. Our results suggest that as long as basal levels of Cx26 expression is maintained within the human population expressing mutations in the *GJB2* gene, mammary gland development and function may be unaffected.

## Supporting Information

Figure S1 **BLG-Cre; Cx26^fl/fl^ mice exhibit a dramatic reduction in Cx26 at parturition and lactation only.** (A) Real-time PCR analysis of mammary glands from control (open columns) and Cre-treated (solid columns) mice revealed a dramatic reduction in Cx26 mRNA levels in lactating mice while no change was observed at other timepoints. Values are mean levels ±SEM. *p<0.05, ***p<0.001. Western blot analysis of Cx26 during pregnancy revealed no significant difference in Cx26 expression at D9.5 and d12.5. Values are mean levels ±SEM. Immunofluorescent analysis of Cx26 (green, arrows) revealed a qualitative reduction in Cx26 at D12.5 of pregnancy in Cx26 knockdown while no change was observed at D9.5 compared to control mice. Hoechst staining denotes the nuclei. Scale bars = 50 µm. N = 4.
(TIF)

Figure S2 **Cx26 knockdown mice have significantly reduced number of Cx30 gap junctions at parturition.** Quantification of 10 arbitrary Cx30-labelled immunofluorescent images per sample at 20X magnification revealed a significant decrease in Cx30 gap junctions compared to control mice at parturition (A) but not at lactation (B). Values are mean levels ±SEM. N = 8.
(TIF)
