# Supplementary figures and images for: Mammary Gland Specific Knockdown of the Physiological Surge in Cx26 during Lactation Retains Normal Mammary Gland Development and Function
Source: PLoS One. 2014 Jul 2;9(7):e101546. doi: 10.1371/journal.pone.0101546 (PMC4079510; doi:10.1371/journal.pone.0101546)

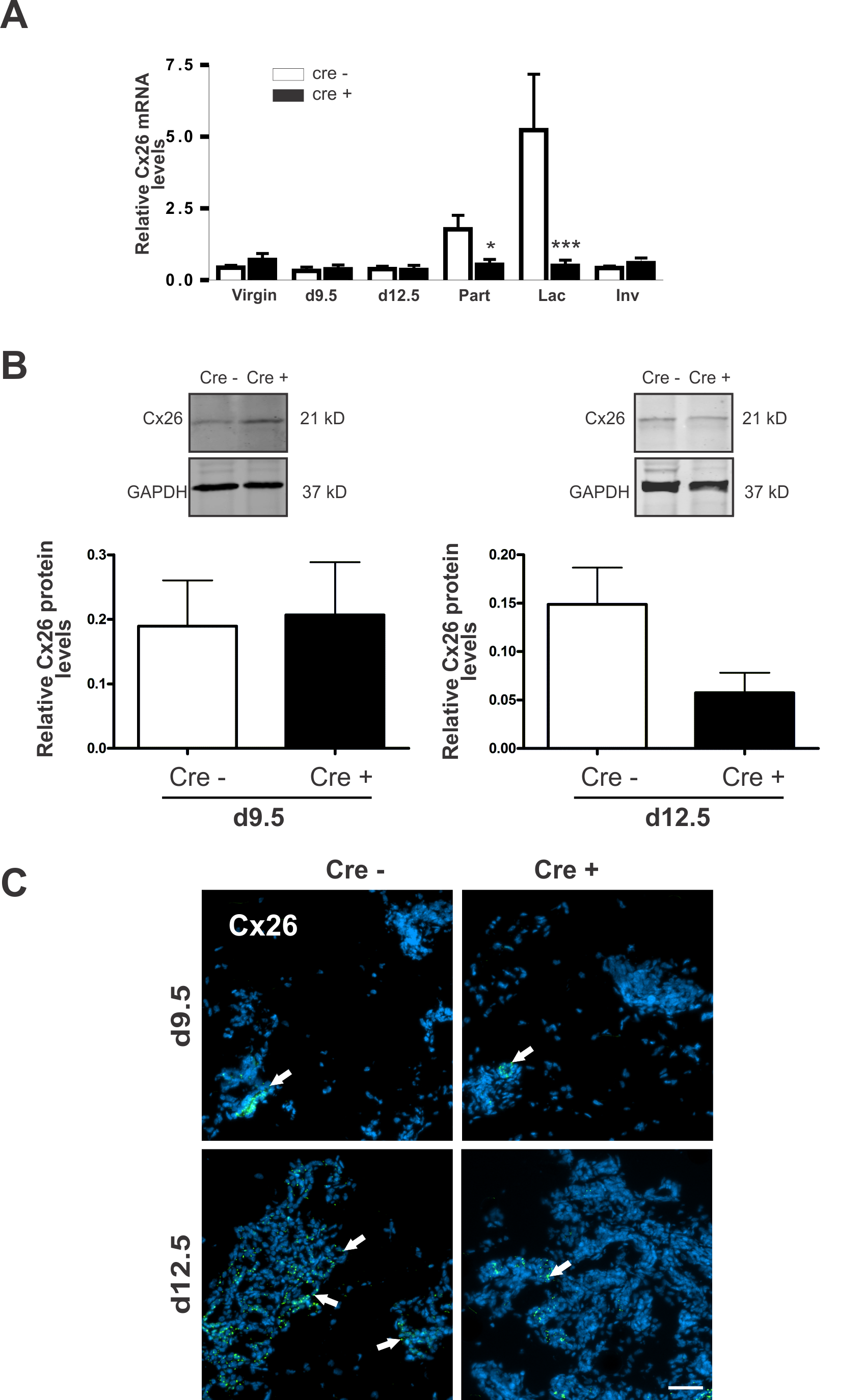

Supplement: Figure S1 — BLG-Cre; Cx26fl/fl mice exhibit a dramatic reduction in Cx26 at parturition and lactation only. (A) Real-time PCR analysis of mammary glands from control (open columns) and Cre-treated (solid columns) mice revealed a dramatic reduction in Cx26 mRNA levels in lactating mice while no change was observed at other timepoints. Values are mean levels ±SEM. *p<0.05, ***p<0.001. Western blot analysis of Cx26 during pregnancy revealed no significant difference in Cx26 expression at D9.5 and d12.5. Values are mean levels ±SEM. Immunofluorescent analysis of Cx26 (green, arrows) revealed a qualitative reduction in Cx26 at D12.5 of pregnancy in Cx26 knockdown while no change was observed at D9.5 compared to control mice. Hoechst staining denotes the nuclei. Scale bars = 50 µm. N = 4. (TIF) [file pone.0101546.s001.tif]

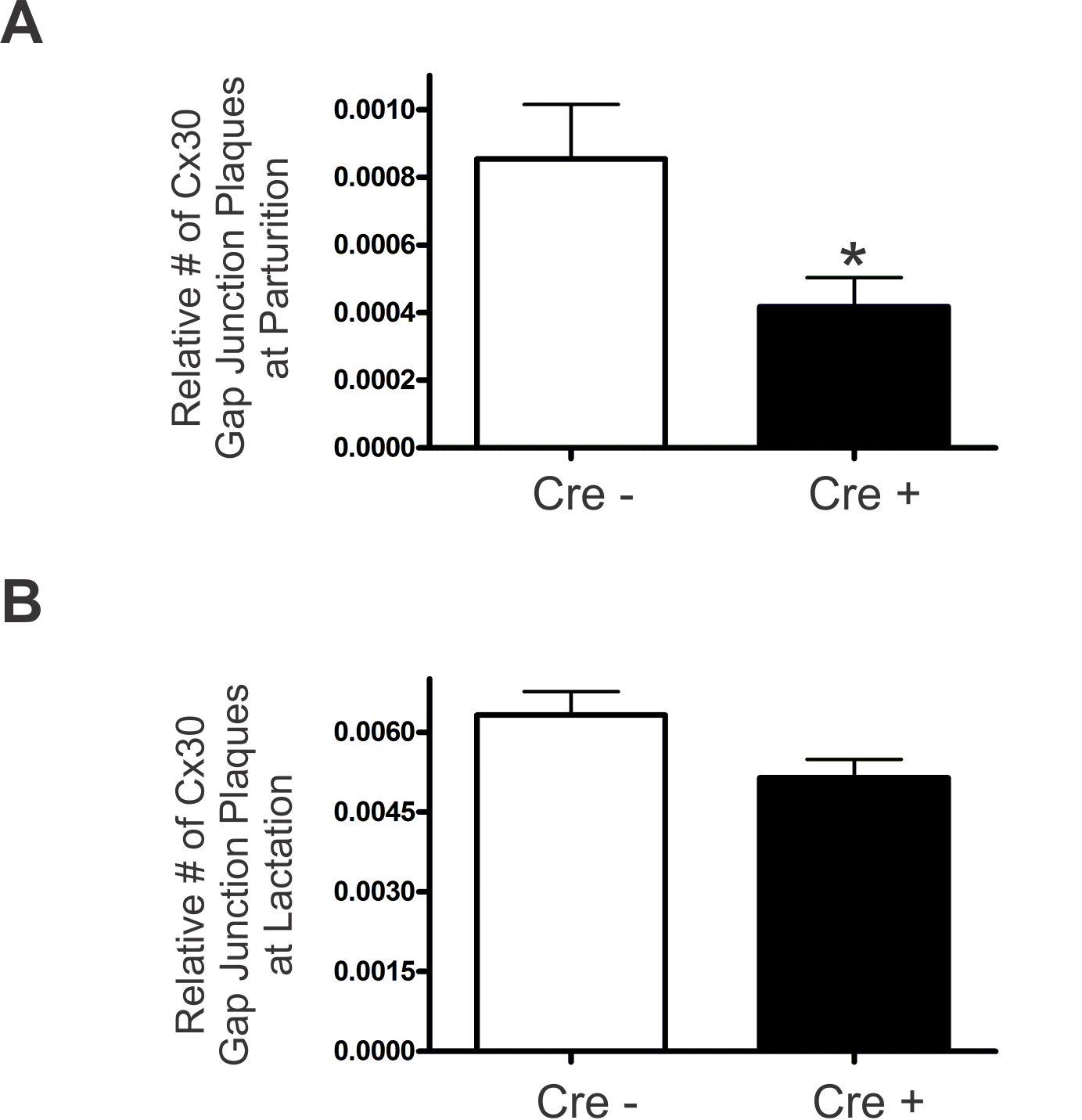

Supplement: Figure S2 — Cx26 knockdown mice have significantly reduced number of Cx30 gap junctions at parturition. Quantification of 10 arbitrary Cx30-labelled immunofluorescent images per sample at 20X magnification revealed a significant decrease in Cx30 gap junctions compared to control mice at parturition (A) but not at lactation (B). Values are mean levels ±SEM. N = 8. (TIF) [file pone.0101546.s002.tif]
